# The role of pre-onset hair hormone in predicting the prognosis of patients with severe pneumonia and acute COVID-19 outbreak

**DOI:** 10.1016/j.heliyon.2024.e30636

**Published:** 2024-05-03

**Authors:** Yuanyuan Jia, Deyi Qi, Tiantian Wang, Yuyao Zhang, Xufeng Chen, Huihua Deng, Dianhuai Meng

**Affiliations:** a Rehabilitation Center, The First Affiliated Hospital with Nanjing Medical University, Nanjing, 210029, Jiangsu, China; b Department of Emergency and Critical Care Medicine, The First Affiliated Hospital with Nanjing Medical University, Nanjing, 210029, Jiangsu, China; c School of Biological Science & Medical Engineering, Southeast University, Nanjing, 211189, Jiangsu, China

**Keywords:** Hair hormone, Progesterone, COVID 19, Severe pneumonia, Prognosis, Mortality

## Abstract

Numerous research works have investigated the potential impact of endocrine hormones on the severity of COVID-19-related pneumonia in individuals. However, there are few studies on the effect of pre-onset neuroendocrine hormones on the prognosis of COVID-19 patients. This study looked into the prognostic value of pre-onset hair hormone levels in COVID-19 infected individuals. This study included 27 patients with COVID-19 and collected patient information and laboratory indicators. The hormone levels in hair were determined by liquid chromatography-tandem mass spectrometry (LC-MS/MS). Within 28 days, 63 % of the patients in this study passed away. With 28-day mortality as the outcome index, urea nitrogen, CURB-65 score and pneumonia severity score (PSI) of 2 groups were statistically significant (*P* < 0.05). Among all hormone levels detected in hair, only progesterone level was substantially correlated negatively with COVID-19 patients' 28-day mortality(*P* < 0.05). The level of progesterone in hair was substantially adversely connected with the death rate at 28 days of COVID-19 patients, according to correlation and logistic regression analysis(*P* < 0.05). Among patients with COVID-19 pneumonia, hair progesterone levels were strongly associated with 28-day mortality, which emphasizes hair progesterone's importance as a prognostic factor.

## 1 Introduction

The coronavirus disease 2019 (COVID-19) is still wreaking havoc on the world's healthcare systems and resulting in horrendous rates of morbidity and death [1]. At the same time, the adverse complications associated with COVID-19 place a tremendous burden of disability [2,3]. SARS Coronavirus 2 (SARS-CoV-2) causes COVID-19 by infecting cells through the ACE2 receptor. This process is initiated by transmembrane serine protease 2 (TMPRSS2) [4]. ACE2 and TMPRSS2 are highly expressed in many endocrine glands [5]. Studies have been conducted on COVID-19 pneumonia and endocrine gland function, although they tend to focus on the function of a single endocrine axis rather than the entire system. A major endocrine target of SARS-CoV-2 could be the hypothalamic-pituitary-adrenal (HPA) axis [6] and the hypothalamic-pituitary-thyroid (HPT) axis [7]. The potential impact of SARS-CoV-2 infection on the hypothalamic-pituitary-gonadal(HPG) axis and consequently male reproductive function is equally concerning [8]. The melatonin system has also been highlighted in COVID-19 pneumonia, where melatonin was found to effectively regulate the immune response and neuroinflammation induced by SARS-CoV-2 [9]. The neuromodulation network known as the endocannabinoid system (ECS) is crucial for controlling a wide range of physiological and immunological functions [10]. Cannabinoids may shield COVID-19 pneumonia patients by preventing the production of cellular inflammatory factors, as demonstrated by PALAND N et al. [11]. "Cytokine storms" seem to be significant mediators of endocrine axis interactions with COVID-19, according to studies [12]. As a result, this study included the aforementioned five axes or systems in order to examine the connection between COVID-19 and endocrine hormones.

Neuroendocrine hormones are commonly measured in blood, urine, saliva and hair. Hormone levels in saliva and blood belong to acute biomarkers, while hormone levels in urine belong to short-term biomarkers. Acute or short-term biomarkers are unstable and susceptible to interference by internal (such as circadian secretion rhythm) and external factors (such as environment, body position, sample collection behavior, etc.) [13]. However, testing hormone levels in hair can overcome these deficiencies. Hormones in the hair can reflect hormone levels over a recent period of time, similar to how hemoglobinA1c(HbA1C) is used to assess serum glucose levels [14]. Additionally, because of the nature of hair growth, the hair from the previous month remains in the scalp. It is this 1 cm section of hair near the scalp that will stabilize the hormones from the month prior to sampling [15]. The hormone level in hair reflects the long-term function of the endocrine axis, which has the advantages of cumulative, persistent, retrospective and non-invasive. This study used these advantages of hair samples to detect hormone levels to explore its role in prognostic assessment of COVID-19 patients [16].

However, using the LC-MS/MS method to detect five axes of the same hair sample is difficult. HPT axis needs to be independently detected, considering that there have been some studies on the correlation between HPT axis and COVID-19 pneumonia [17,18]. This study therefore covered four of the five common endocrine axes and selected appropriate representative hormones for each axis.

## 2 Materials and methods

### 2.1 Participants and hair collection

This study analyzed all patients newly admitted to the emergency intensive care unit (EICU) of the First Affiliated Hospital with Nanjing Medical University who were infected with COVID-19 between December 20, 2022, and January 20, 2023. A written informed consent was signed by patients or their guardians prior to enrollment. Some patients were excluded either because they had used hormone therapy within the past month or because their hair did not meet the testing requirements. A total of 56 patients in two units (37 beds) of the EICU were screened. Out of these, 12 patients chose not to participate, 6 patients received hormone therapy, and 11 patients had insufficient hair length. This study ultimately included 27 patients in total. The Ethics Committee gave its approval to the study, which followed the Declaration of Helsinki's guidelines.

Hair samples were collected from all subjects on admission. Because hormone levels in hair are relatively stable, there is no strict requirement for a specific sampling time of day or sampling position. Hair samples were collected by cutting 1 cm of hair from the occipital region, located at the back of the subject's head near the scalp. The hair samples were then placed in a dry ziplock bag, labeled, and stored in a standard refrigerator.

Inclusion criteria:

① Adults;
②Participants in the study included EICU patients who had COVID-19 pneumonia symptoms and positive PCR results;
③ Hair longer than 1 cm can be cut off from the back of the pillow.

Exclusion criteria:

① Individuals with a history of endocrine system disease or steroid use within 1 month prior to infection;
② Dyed or permed hair;
③ Pregnant women.

### 2.2 Detections and assessments

**Laboratory tests:** White blood cell counts, Lymphocyte counts, C-reactive protein, D-dimer, Urea nitrogen, Procalcitonin.

**Scales:** CURB-65 scores, Pneumonia severity index (PSI).

**Axis and system:** HPA axis: Cortisol(F), Cortisone(E), Dehydroepiandrosterone (DHEA);

HPG axis: Testosterone(T), Progesterone(P);

ECS:1-Arachidonic acid glycerol(1-AG), Anandamide (AEA);

The melatonin system: Melatonin (MEL), N-Acetylserotonin (NAS).

### 2.3 Determination of the hormones in hair

The hormones in hair were simultaneously detected using high-performance liquid chromatography-tandem mass spectrometry (LC-MS/MS), where an API 3200 Q-TRAP mass spectrometer (Applied Biosystems, Inc., USA) was combined with an Agilent 1200 liquid chromatograph (Agilent Technologies, Inc., USA), following a previously developed method [16]. Hair samples were rinsed with 5 mL of methanol for 2 min, and then heated to 50 °C for drying. The washing procedure was repeated twice in total. In a 2 mL centrifuge tube, hair samples were weighed after measuring 1–2 mm in length and cut into powder. After incubation in methanol for 24 h at 25 °C, the mixture was centrifuged at 1.2 × 104 r/min for 5 min. Once the supernatant was transferred to another clean centrifuge tube, it was evaporated using nitrogen at 40 °C. The residue was then redissolved in 50 mL of mobile phase for LC-MS/MS analysis.

There was good performance, with limits of detection and quantification of 0.2 and 0.5 pg/mg for F, 0.3 and 1.1 pg/mg for E, 1.4 and 4.6 pg/mg for DHEA, 0.7and 2.2 pg/mg for T, 0.2 and 0.5 pg/mg for P, 0.3 and 0.1 pg/mg for AEA, 0.6 and 1.7 pg/mg for 1-AG, 0.08 and 0.25 pg/mg for MEL, and 0.5 and 1.4 pg/mg for NAS, respectively. The recovery, intra-day, and inter-day coefficients of variation met the requirements.

### 2.4 Statistical analysis

SPSS 22.0 software was used for data analysis. The Shapiro-Wilk method was used to examine the distribution types of all measured data. The majority of the data in this study were skewed, the Mann-Whitney U rank sum test was used to compare data between groups, and the data were reported as median and interquartile range (M, IQR). The Count information is expressed as frequency and percentage[n(%)], and the χ2 test was performed to compare the groups. Receiver operating characteristics (ROC) curve analysis was utilized to ascertain the crucial value of hair hormone levels, with a *P* < 0.05 level regarded statistically significant.

## 3 Results

The study comprised 27 patients in all who tested positive for COVID-19 by PCR. 79 years old was the median patient age (IQR: 71–83). There were 9 (29.6 %) females in the study. Patients were grouped according to death or survival within 28 days, and statistical analysis of baseline information for the two groups was shown in Table 1. There were no significant differences in gender, age, BMI, smoking, drinking, hypertension, diabetes, coronary heart disease and cerebral infarction between the two groups (*P* > 0.05).

**Table 1:** Statistical analysis of basic information of dead and surviving patients [M(IQR)/n(%)].

Basic information	Die within 28 days(n = 17)	Survive within 28 days(n = 10)	Z/*χ*^2^	*P* value
Gender				
male	13(76.47%)	6(60.00%)	0.819	0.415
female	4(23.53%)	4(40.00%)		
Age(years)	82.00(2.00)	60.00(12.00)	1.586	0.115
BMI(kg/m^2^)	29.39(0.15)	27.55(6.95)	0.251	0.824
HT				
yes	12(70.59%)	5(50.00%)	1.144	0.415
no	5(29.41%)	5(50.00%)		
DM				
yes	5(29.41%)	5(50.00%)	1.144	0.415
no	12(70,59%)	5(50.00%)		
CAD				
yes	5(29.41%)	2(20.00%)	0.290	0.678
no	12(70,59%)	8(80.00%)		
Stroke				
yes	5(29.41%)	4(40.00%)	0.318	0.683
no	12(70,59%)	6(60.00%)		

HT: Hypertension, DM: Diabetes, CAD: Coronary Artery Disease.

Mann-Whitney u test was used to analyze the relevant information (leukocyte, lymphocyte, C-reactive protein, D-dimer, urea nitrogen, procalcitonin, curb-65, and PSI) of the 2 groups. There were statistically significant differences in urea nitrogen, CURB-65 score and PSI between dead and surviving patients within 28 days (*P* = 0.027, *P* = 0.018, *P* = 0.003)[Table 2].

**Table 2:** Statistical analysis of relevant information of dead and surviving patients [M(IQR)].

Relevant information	Die within 28 days(n = 17)	Survive within 28 days(n = 10)	Z	*P* value
leukocyte (10^9/L)	9.74(7.63)	9.90(10.58)	−0.703	0.505
lymphocyte (10^9/L)	0.42(0.51)	0.78(0.74)	−1.659	0.103
CRP (mg/L)	56.54(46.24)	50.2(46.72)	0.050	0.960
D-dimer (mg/L)	2.91(6.19)	1.62(9.98)	0.276	0.786
UA (mmol/L)	15.12(11.99)	8.87(7.35)	2.209	0.027
PCT (ng/mL)	0.26(1.33)	0.17(0.85)	1.206	0.243
PSI	169.00(39.50)	112.00(58.25)	2.865	0.003
CURB-65	3.00(1.50)	2.00(2.00)	2.443	0.018

PSI:pneomonia severity index, PCT:procalcitonin, UA:urea nitrogen, C-reactive protein:CRP.

Mann-Whitney u test was used to analyze the levels of 9 hormones (F, E, DHEA, T, P, AEA, 1-AG, MEL, NAS) in the hair of the two groups of patients. The level of progesterone in hair was significantly different between dead and surviving patients (*P* = 0.023)[Table 3].

**Table 3:** Statistical analysis of hormone levels in hair of dead and surviving patients [M(IQR)].

hormones(pg/mg)	Die within 28 days(n = 17)	Survive within 28 days(n = 10)	Z	*P* value
F	87.40(113.35)	81.50(115.03)	−0.653	0.537
E	74.05(75.42)	77.95(129.93)	−1.507	0.141
DHEA	12.25(12.88)	15.65(12.75)	−0.753	0.473
T	4.05(2.45)	4.70(2.20)	0.503	0.639
P	3.90(2.03)	7.25(4.30)	−2.235	0.023
AEA	22.55(14.28)	12.8(9.67)	1.432	0.155
1-AG	61.00(38.72)	59.80(73.95)	−0.050	0.980
MEL	0.81(0.61)	0.77(0.64)	−0.326	0.749
NAS	31.45(21.55)	25.30(21.20)	0.753	0.473

F:cortisol, E:cortisone, DHEA:dehydroepiandrosterone, T:testosterone, P:progesterone, AEA:Anandamide, 1-AG:1-Arachidonic acid glycerol, MEL:melatonin, NAS:N-Acetylserotonin.

The Spearman correlation analysis showed significant associations of UA, CURB-65, PSI and P level in hair with the death rate at 28 days in COVID 19 Pneumonia patients(*P* = 0.024, *P* = 0.011, *P* = 0.002, *P* = 0.022, respectively)[Table 4].

**Table 4:** Correlation between 28-day mortality scores and variables.

		UA	CURB-65	PSI	P
28-day mortality	r	0.433	0.479	0.562	−0.483
	*p* value	0.024	0.011	0.002	0.022
	n	27	27	27	27

Spearman correlation analysis.

Logistic analysis revealed that among COVID-19 Pneumonia patients, UA, CURB-65 scores, PSI, and P level in hair were significant predictors of 28-day death(*P* = 0.045, 0.024, 0.009, 0.033 respectively)[Table 5].

**Table 5:** Logistic analysis between 28-day mortality scores and variables.

Variables	B	S.E.	Wald	df	*Sig.*	95 % CI	
						lower	upper
UA	0.175	0.087	4.009	1	0.045	1.004	1.413
CURB-65	1.296	0.576	5.063	1	0.024	1.182	11.306
PSI	0.049	0.019	6.836	1	0.009	1.102	1.089
P	−0.506	0.238	4.523	1	0.033	0.378	0.961

Logistic regression analysis.

**Fig. 1:**
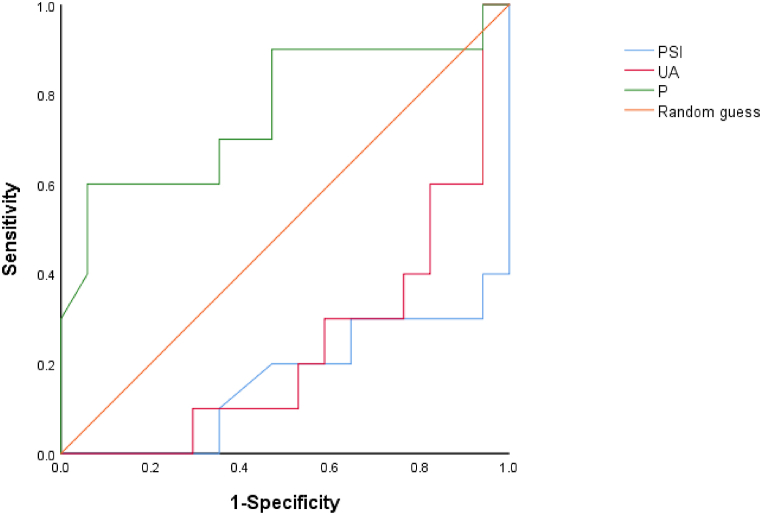
The receiver operating characteristic (ROC) curve illustrating the predictive ability of P against mortality. Note: Area under ROC curve = 0.762.

In ROC curve analyses for identifying predictors of mortality, the area under the curve (AUC) for the P was 0.762 (*P* = 0.025); for the UA, the AUC was 0.241 (*P* = 0.027); and for the PSI, the AUC was 0.165 (*P* = 0.004) [Fig. 1].

A 28-day mortality cutoff value of 7.05 pg/mg had a 94.1 % specificity and 60.0 % sensitivity. With 76.2 % accuracy, P ≤ 7.05 pg/mg predicted the 28-day mortality rate in critically ill COVID-19 pneumonia patients.

## 4 Discussion

The study's findings indicated that both of most typical COVID-19 patient symptoms were fever and cough. This result is consistent with previous studies [19,20]. In this study, 74 % of the patients the symptoms of dyspnea, possible reason is that we recruited patients from intensive care unit.

Research has shown that when a patient suffers from community-acquired pneumonia, the CURB-65 and PSI scores can reliably predict the patient's death [21,22]. The PSI and CURB-65 scores are helpful in determining the severity of COVID-19 pneumonia and are dependable indicators of mortality, according to numerous studies [23]. We found that the PSI and CURB-65 scores were significant predictors of 28-day death in individuals with COVID-19 pneumonia.

Only progesterone levels demonstrated a significant inverse relationship with mortality among the nine hair hormones that were found. This means that patients may be more likely to die if their progesterone levels are lower. The HPG axis is composed of hypothalamus, pituitary gland and gonad. The hypothalamus releases gonadotropin to the pituitary gland, which releases gonadotropin to the gonad gland, and female ovaries and male testis secrete sex hormone, participating in feedback and negative feedback to regulate human reproductive behavior and sexual behavior. The abnormal amount of progesterone in both sexes is probably a result of impairment of the ovary, adrenal, or hypothalamuspituitary axis. While the prevalence is the same for men and women, a sex difference appears in case mortality (deaths/reported cases), with almost twice as many men with COVID-19 suffering from severe symptoms or dying compared to women [24,25]. MAUVAIS-JARVIS F et al. [26] showed that the risk of serious COVID-19 outcomes was consistently lower in women than in men, suggesting that biological sex plays an important role in protecting women [27]. DING T.et al. showed that the severity of COVID-19 was negatively correlated with estradiol (E2), suggesting that it may be a protective factor [28]. The possible explanation for this correlation is that E2 regulates cytokines related to inflammation and immunity [29], which are important mediators of the effects of the endocrine axis related to COVID-19. Studies have shown that progesterone is an important immunomodulatory and anti-inflammatory hormone [30], which promotes lung tissue repair by up-regulating the expression of epidermal growth factor dual regulatory protein in the lung [31]. GORDON D.E. et al. indicates that progesterone also inhibits the replication of the SARS-CoV-2 virus in infected cells [32].

In our investigation of individuals with COVID-19 pneumonia, the progesterone level of their hair was the best predictive of 28-day mortality. Simultaneously, we computed that in individuals with severe COVID-19 pneumonia, progesterone levels in hair predicted 28-day mortality at a cutoff of 7.05 pg/mg. It is strongly advised to take the progesterone level into account while estimating the prognosis for COVID-19 pneumonia.

The innovation of this study is that it is the first to explore the influence of preonset neuroendocrine hormones on the results of COVID-19 patients. Meanwhile, this study is not without limitations. First, there could be sampling error due to the small sample size in this study. Between December 2022 and January 2023, when China was experiencing severe outbreaks of COVID-19, the study sample was gathered. During this period, the patients' conditions were rapidly changing. All critically ill COVID-19-positive patients who satisfied the criteria during this time frame were included in the study. Secondly, the study's sample may be typical of senior Chinese patients suffering from severe COVID-19 infection. Consequently, it's critical to take consistency with other age groups, geographic areas, and ethnic groups into account when extrapolating the results. But as of right now, there's no proof that the findings' extrapolation is irrational.

## 5 Conclusion

There was a strong correlation between chronically low progesterone levels in hair and 28-day mortality in patients with COVID-19 pneumonia in this small sample study.

## Statement of ethics

This study was reviewed and approved by the Ethics Committee of Jiangsu Provincial People's Hospital (2022-SR-553). All participants provided written informed consent to participate in the study.

## Funding

This work was supported by the Key Research & Development Program of 10.13039/501100002949Jiangsu Province (Grant No. BE2021012-4).

## Data availability

Data will be made available on request.

## CRediT authorship contribution statement

**Yuanyuan Jia:** Writing – original draft, Formal analysis, Data curation. **Deyi Qi:** Writing – original draft, Visualization. **Tiantian Wang:** Methodology. **Yuyao Zhang:** Writing – original draft. **Xufeng Chen:** Resources. **Huihua Deng:** Resources. **Dianhuai Meng:** Writing – review & editing, Conceptualization.

## Declaration of competing interest

The authors declare the following financial interests/personal relationships which may be considered as potential competing interests: Dian huai Meng reports financial support was provided by the Key Research & Development Program of Jiangsu Province (grant No. BE2021012-4). If there are other authors, they declare that they have no known competing financial interests or personal relationships that could have appeared to influence the work reported in this paper.
